# Antitumor effects of volatile components of *Magnolia salicifolia* essential oil against breast cancer cells are mediated via mitochondrial apoptosis

**DOI:** 10.3389/fonc.2026.1917503

**Published:** 2026-08-31

**Authors:** Takuya Nagata, Tadaaki Satou, Kenjiro Ishii, Aya Sasaki, Prabodh Satyal, Manabu Watanabe, Brannick Riggs, Yoshihisa Saida

**Affiliations:** 1 Department of Surgery, Toho University Ohashi Medical Center, Tokyo, Japan; 2 Department of Pharmaceutical Sciences, International University of Health and Welfare, Narita, Japan; 3 Aromatic Plant Research Center, Lehi, UT, United States; 4 Prime Meridian Healthcare, Pleasant Grove, UT, United States

**Keywords:** apoptosis, breast cancer, citral, essential oil, magnolia salicifolia, volatile compounds

## Abstract

**Background:**

Plant essential oils exhibit anticancer properties. However, the antitumor effects of *Magnolia salicifolia* essential oil remain unexplored.

**Methods:**

We evaluated the antitumor activities of *M. salicifolia* essential oil and its volatile components in 4T1 murine breast cancer cells. Cell viability was assessed using the MTT assay. The concentration of the volatile components was analyzed using gas chromatography (GC)-mass spectrometry (MS). Morphological changes in the cells were examined using fluorescence microscopy. The underlying mechanisms regarding the antitumor effects of *M. salicifolia* essential oil were investigated using western blotting.

**Results:**

*M. salicifolia* essential oil exhibited potent antitumor activity with a Half maximal inhibitory concentration (IC50) of 1.05 µg/mL in 4T1 cells. The antitumor effects of the volatile components decreased in a distance-dependent manner. GC-MS analysis revealed that the oil contained high levels of citral (neral + geranial), 1,8-cineole, and transanethole. Citral demonstrated significantly stronger antitumor activity than 1,8-cineole (IC50 = 1.93 vs. 68.4 µg/mL; n=3 independent experiments per condition). *In vivo*, inhalation of *M. salicifolia* essential oil vapor significantly reduced tumor incidence (7 of 15 mice, 47% vs. 14 of 15 mice, 93% in controls; Fisher’s exact test, p=0.0142) and tumor area and weight (n=15 mice/group, p<0.005), without affecting body weight. Mechanistically, citral induced apoptosis via mitochondrial dysfunction, characterized by a decrease in membrane potential, increased cytochrome c levels, and activation of caspase-3 and caspase-9. Comparable antitumor activity was confirmed in the human breast cancer cell line MCF-7, with IC50 values of 1.021 µg/mL for the essential oil and 1.285 µg/mL for citral, supporting the translational relevance of these findings. In addition, *M. salicifolia* essential oil exhibited preferential cytotoxicity toward 4T1 cancer cells over the non-tumorigenic MCF-10A mammary epithelial cell line, indicating cancer-selective activity.

**Conclusion:**

The volatile components of *M. salicifolia* essential oil exert strong antitumor effects primarily through citral-mediated mitochondrial apoptosis. These findings highlight the potential of inhaled phytochemicals as novel anticancer agents.

## 1 Introduction

Plant-derived essential oils have attracted considerable attention as potential anticancer agents because they often exhibit multiple biological activities with relatively low toxicity. Among them, essential oils are complex mixtures of volatile low-molecular weight compounds that possess antimicrobial, antioxidant, anti-inflammatory, and anticancer properties (1). Several essential oils, including frankincense (*Boswellia carteri*) (2, 3), lavender (*Lavandula officinalis*), thyme (*Thymus vulgaris*) and oregano (*Origanum vulgare*), have been reported to inhibit proliferation and induce apoptosis in various cancer cell types, including breast, gastoric, colorectal, liver, and skin cancers (4). These findings suggest that volatile phytochemicals may represent a promising source of novel anticancer compounds.

Among the numerous constituents of essential oils, citral (3,7-dimethyl-2,6-octadienal; a mixture of neral and geranial) has emerged as one of the most potent anticancer monoterpenoids. Citral is abundant in citrus fruits, lemongrass (*Cymbopogon citratus)*, lemon myrtle (*Backhousia citriodora)*, litsea (*Litsea cubena)*, and several medicinal plants (5). Previous studies have demonstrated that citral suppress proliferarion and induces apoptosis in breast, gastric colorectal, ovarian, uterine, and prostate cancer cells through activation of mitochondrial apoptotic signaling (6, 7). In addition, citral inhibits cancer stem cell populations (8, 9) by suppressing aldehyde dehydrogenase (ALDH) activity and Wnt signaling (10, 11), suggesting that its biological activity extends beyond simple cytotoxicity. Consistent with these observations, our previous studies demonstrated that citral-rich essential oils, including lemon myrtle, lemongrass, litsea, and melissa, exhibit potent growth-inhibitory activity against breast cancer cells (12).

Unlike conventional chemotherapy, essential oils possess an important characteristic: many of their active constituents are volatile and can therefore be administered by inhalation. Inhaled volatile phytochemicals are capable of reaching the alveolar epithelium (13), where a fraction may be absorbed into the systemic circulation before being metabolized (14, 15). This unique pharmacological property raises the possibility that inhaled essential oils could exert biological effects not only through olfactory stimulation but also through direct systemic delivery of bioactive compounds (16). Aromatherapy has therefore attracted increasing interest as a complementary approach for cancer care. However, despite this potential, most previous studies have investigated only the direct application of essential oils or isolated compounds to cultured cancer cells (17, 18). Experimental evidence demonstrating whether volatile components alone can inhibit tumor growth, particularly under inhalation-mimicking conditions or *in vivo*, remains limited.

*Magnolia salicifolia* Maxim., a medicinal tree endemic to Japan (19, 20), produces an essential oil characterized by a pleasant fragrance and a unique composition rich in monoterpenes, including citral, 1,8-cineole, and trans-anethole. The dried flower buds of *M. salicifolia* have long been used in traditional Japanese herbal medicine (Shin’i) for the treatment of rhinitis, sinusitis, headache, and dizziness (21). Although the chemical composition of its essential oil has been reported previously, its potential anticancer activity, particularly that of its volatile constituents, has not yet been investigated.

Therefore, the present study was designed to address these knowledge gaps. Specifically, we investigated the antitumor activity of *M. salicifolia* essential oil and its volatile components against murine breast cancer cells using a novel distance-dependent volatile exposure model. We further identified the principal active constituents by GC-MS analysis, examined the contribution of citral to the observed biological activity, evaluated the antitumor effects of inhaled volatile compounds in a murine breast cancer model, and explored the underlying molecular mechanism focusing on mitochondria-mediated apoptosis. By combining *in vitro*, volatile exposure, and *in vivo* approaches, this study provides experimental evidence supporting the potential of inhalable phytochemicals as candidates for future anticancer therapeutic development.

## 2 Materials and methods

### 2.1 Essential oil preparation

The essential oil of *M. salicifolia* was obtained from Oak Village Co., Ltd. and Hida Sangyo Co., Ltd. (Takayama City, Gifu Prefecture, Japan) (origin: Japan; extraction method: steam distillation). This product was purified using a conventional steam distillation method, and its components were identified using gas chromatography-mass spectrometry (GC-MS). Citral (a *cis*- and *trans*- mixture) and 1,8-cineole (used as standards) were both purchased from Fujifilm Wako Pure Chemical Industries, Ltd. (Japan), dissolved in Dimethyl sulfoxide (DMSO) to a concentration of 10⁻² g/mL, and stored at –20°C until further analyses.

### 2.2 Cell culture and viability assay

The mouse mammary cancer cell line 4T1, the human breast cancer cell line MCF-7 and the non-tumorigenic human mammary epithelial cell line MCF-10A were obtained from the American Type Culture Collection (USA). Briefly, 4T1 and MCF-7 cells were cultured in Dulbecco’s modified Eagle’s medium (DMEM) (Invitrogen Corporation, USA) supplemented with 5% fetal bovine serum (Cytiva Inc., Japan). MCF-10A cells were cultured in mammary epithelial cell growth media supplemented with 5% fetal bovine serum, 10 ng/mL EGF, 0.5 µg/mL hydrocortisone, and 5 µg/mL insulin (Promo Cell GmbH, Heidelberg, Germany). Antibiotics and antifungal agents (streptomycin and amphotericin B; Thermo Fisher Scientific Inc., USA) were added to all culture media. MCF-7 cells were treated with *M. salicifolia* essential oil and citral following the identical protocol used for 4T1 cells. Cell viability was assessed by MTT assay, and morphological analysis by fluorescent microscopy.

### 2.3 Volatile exposure model

**Figure 1:**
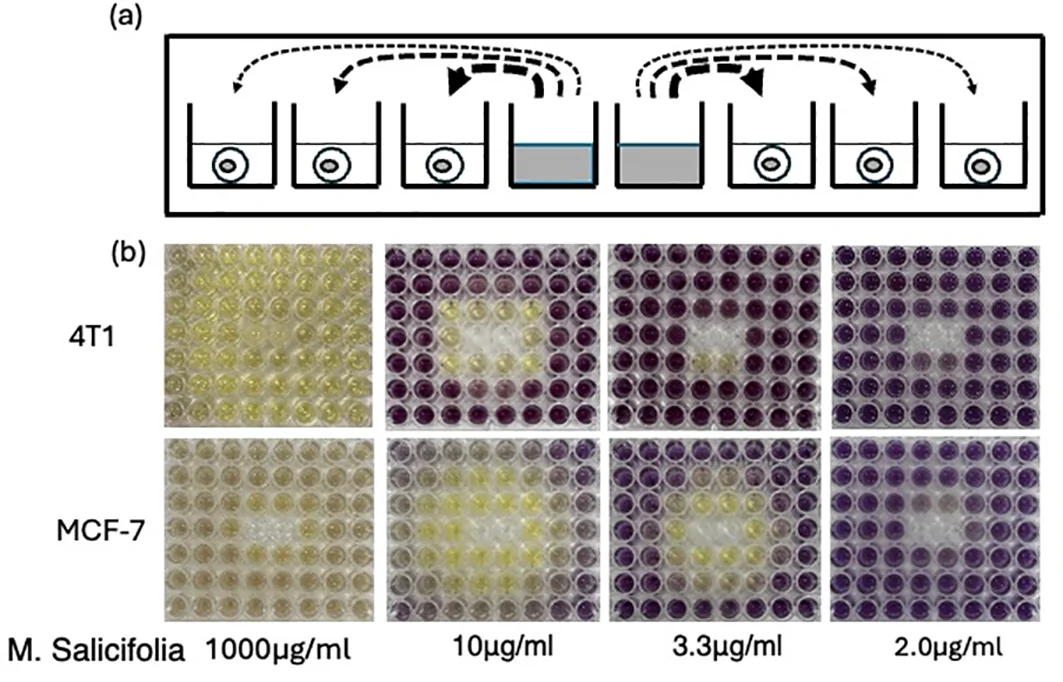
Schematic diagram and antitumor effects showing the movement of volatile components of *Magnolia salicifolia*. **(a)** *M. salicifolia* essential oil was placed in the center of a 96-well plate, and 4T1 mouse mammary cancer cells were seeded in the wells around it. Each well was connected by an upper space so only the volatile components could reach the other wells. Dashed lines showed the movement of volatile components of *M. salicifolia*. **(b)** The antitumor effects of the components of *M. salicifolia* essential oil to the 4T1 and MCF-7 cells were investigated using an MTT assay. DMSO (100%) served as a control. *M. salicifolia* essential oil was used at the original concentration (1000 µg/mL), diluted 100-fold (10 µg/mL), 300-fold (3.3 µg/mL), and 500-fold (2.0 µg/mL). Fifty microliters of each dilution was added to the two central wells in each experiment (n=3, biological replicates).

*M. salicifolia* essential oil was placed in the center of a 96-well plate, and 4T1 cells, MCF-7 or MCF-10A were placed around it for exposure to its volatile components (**Figure 1a**). Each well was connected by an upper space, allowing only the volatilized gas to reach the other wells. Exposure plates were maintained in a standard cell culture CO2 incubator (37 °C, 5% CO2, with the incubator’s standard humidification); no additional forced air exchange or independent humidity control specific to the exposure system was applied. Citral concentration in each well was measured as a single endpoint after the full 48 h incubation period (see GC-MS analysis); time-resolved evaporation kinetics during the incubation period were not assessed. The antitumor effects of the essential oil components were quantified using a 3-(4,5-dimethylthiazol-2-yl)-2,5-diphenyltetrazolium bromide (MTT) assay. Briefly, cancer cells were seeded in a 96-well plate at a density of 1×10^4^ cells/100 µL. *M. salicifolia* essential oil was administered to the cultured cells at concentrations of 6×10–^5^ to 2.0 µg/mL and cultured at 37°C for 48 h. For the volatile component experiment, 50 µL of essential oil with varying concentrations, ranging from a 1× stock solution (1.0 mg/mL) to a 1:500 dilution (2.0 µg/mL), was administered to the two central wells and allowed to react for 48 h at 37°C. After the reaction, 10 µL of Solution I from the MTT Cell Proliferation Assay Kit (Roche Diagnostics, Switzerland) was added to each well. After incubation at 37°C for 4 h, 100 µL of the visualization solution was added to each well and incubated overnight at 37°C. The absorbance was then measured at 570 nm using a multiplate reader (iMark plate reader; Bio-Rad Laboratories, Hercules, CA, USA). The wells were defined as “proximal” (0–0.8 cm), “middle” (0.8–1.6 cm), or “distal” (1.6–2.4 cm) depending on their distances from the well containing the essential oil. To evaluate the cancer selectivity of the volatile components, the same distance-dependent exposure protocol was applied in parallel to 4T1 cells and the non-tumorigenic human mammary epithelial cell line MCF-10A, and cell survival was compared between the two cell lines at each well position (proximal, middle, distal).

### 2.4 GC-MS analysis

In this experiment, 0.1 mL of DMEM was dispensed into each well of a 96-well plate. A MonoTrap RCC18 (GL Sciences Co., Ltd., Japan) was then placed in the proximal, middle, and distal wells. Next, 50 µL of *M. salicifolia* essential oil, diluted to 3.3 µg/mL with DMSO, was dispensed into each of the two central wells of the plate and incubated at 37°C for 48 h. Each MonoTrap was then sealed in a glass container containing 2 mL of hexane. The citral concentration in each hexane solution was measured using a GC-MS-QP2010 system (Shimadzu Corporation, Kyoto, Japan). A DB-5MS capillary column (30 m × 0.25 mm I.D., 0.25 µm, nonpolar column, Agilent Technologies, Inc., Japan) was used for the analysis. Helium (99.99995% purity, 1.82 mL/min) was used as the carrier gas. The inlet line and source temperatures were set to 250°C. The column oven temperature gradient was as follows: 40°C for 2 min, then increased to 200°C at a rate of 5°C/min, and finally maintained at 200°C for 2 min. The constituents of each essential oil were analyzed using Gas chromatography–mass spectrometry (GC-MS) at the Aroma Plant Research Institute (Lehi, Utah, USA). Components present at proportions of 5% or more were included in the study. Our MonoTrap adsorption coupled with GC-MS analysis is consistent with recently developed spatial metabolomics methodologies for quantitative evaluation of volatile metabolites across diffusion gradients, supporting the reliability of distance-dependent volatile compound analysis (22).

### 2.5 Morphological analysis

The antitumor effects of the essential oil components and volatile essential oil components on breast cancer cell lines were evaluated by observing individual cells under a fluorescence microscope. Breast cancer cells treated with the essential oils were double-stained with calcein-acetoxymethyl (AM) and propidium iodide (PI) using a cell double-staining kit (Dojindo Laboratories, Inc., Japan). Live cells were stained green, and dead cell nuclei were stained red. After staining, the breast cancer cells were observed using a fluorescence microscope (CKX53; Olympus Corporation, Tokyo, Japan), and the changes were photographed.

To observe citral-induced changes in breast cancer cells, the cells were photographed over time using a light microscope (200× magnification), and their morphological changes were examined. In addition, a field of breast cancer cells was photographed over time using a light microscope (100× magnification), and the effect of citral treatment on cell movement was evaluated.

### 2.6 Annexin V/PI apoptosis assay

4T1 and MCF-7 cells were exposed to M. salicifolia essential oil vapor for 24 h and stained using Annexin V-FITC apoptosis detection kit (Dojindo Laboratories, Kumamoto, Japan). The percentage of Annexin V- and/or PI-positive cells was determined by fluorescence microscopy.

### 2.7 Mitochondrial membrane potential (JC-1) assay

Mitochondrial membrane potential was assessed using the JC-1 MitoMP Detection Kit (Dojindo Laboratories, Kumamoto, Japan). Cells were incubated with JC-1 following treatment with M. salicifolia essential oil, and the shift from red (aggregate, high membrane potential) to green (monomer, depolarized) fluorescence was evaluated by fluorescence microscopy.

### 2.8 Intracellular ROS assay

Intracellular reactive oxygen species (ROS) generation was measured using ROS Assay Kit -Highly Sensitive DCFH-DA (Dojindo Laboratories, Kumamoto, Japan). Cells treated with M. salicifolia essential oil were loaded with the probe, and fluorescence intensity was measured by fluorescence microscopy.

### 2.9 Establishment of an *in vivo* murine metastatic breast cancer model

This study was approved by the Toho University Animal Experiment Committee (Approval code: Dosho 23-552). Briefly, 30 4-week-old female BALB/c mice (BALB/cAnNCrlCrlj; Charles River Japan Co., Ltd., Yokohama, Japan) were housed in six cages, with five mice per cage. The mice were provided with free access to food and water throughout the experiment. Before treatment, the mice were sedated with intraperitoneal administration of anesthetic preparations (Dexmedetomidine Hydrochloride 0.3mg/kg; Kyoritsu Seiyaku corp. Tokyo, Japan, Midazolam 4.0mg/kg; Maruishi Parmceutical. Co. Osaka, Japan, Butorphanol 5.0mg/kg; Meiji Animal Health Co. Tokyo, Japan), and 5×10^5^ 4T1 cells were transplanted subcutaneously into the back of each mouse. Mice were randomly allocated to the MS or control group (15 mice each) prior to initiation of the exposure period. Humane endpoints were defined in accordance with the approved animal experimentation protocol (Dosho 23-552), and animals were monitored regularly throughout the study for signs of distress, with early euthanasia performed if protocol-defined criteria were met. Outcome assessment (tumor detection and measurement) was performed by investigators who were not blinded to group allocation. In the MS group, three cages containing five mice each were placed in sealed containers large enough to allow for adequate ventilation and were housed for 8 h per day. Five drops of *M. salicifolia* essential oil were then placed on a dish in a container away from the mouse cages so that the mice could inhale the naturally evaporating gases. In the control group, the mice were kept in containers similar to those in the MS group, but without the presence of *M. salicifolia* essential oil for 8 h per day. After 4 weeks, the mice in both groups were euthanized by cervical sprain that was performed by the skilled professional under the anesthesia with isoflurane (Pfizer, NY, USA), and their body weights were compared. The tumors on the backs of the mice were removed, and the tumor area and weight were compared. Tumor area and weight were measured once, at the time of excision at the end of the 4-week study period; serial in-life tumor measurements were not performed.

### 2.10 Western blotting

For protein extraction, 1×10^7^/10mL of 4T1 cells were treated with citral (*cis*- or *trans*-; 0.19 µg/mL or 1.9 µg/mL, respectively) at 37°C for 14 h. Then, 1 mL of RIPA buffer supplemented with 10 µL of protease inhibitor (Nakalai Tesque Co., Ltd., Japan) was added to the cells, and the mixture was allowed to stand on ice for 5 min. After centrifugation at 14,000×*g* for 15 min, the supernatant was collected to obtain total protein. The protein concentration in the solution was measured using a TAKARA BCA Protein Assay Kit (Takara Holdings Co., Ltd., Japan). Each protein solution was separated via sodium dodecyl sulfate-polyacrylamide gel electrophoresis (SDS-PAGE) and transferred onto a polyvinylidene difluoride (PVDF) membrane using a Trans-Blot Turbo Transfer System (Bio-Rad Laboratories). A pre-stained protein molecular weight marker (Precision Plus Protein Kaleidoscope Standards, Bio-Rad Laboratories) was loaded alongside samples on each gel to enable estimation of protein molecular weight. The membranes were then incubated with the following antibodies for 10min, and washed 4 times with TBS-T(Tris Buffered Saline with Tween^®^20) using the SNAP i.d.2.0 protein detection system: rabbit anti-p53 (PharMingen Corporation, USA), rabbit anti-p-27 (PharMingen), rabbit anti-p-21 (PharMingen), rabbit anti-Bcl-2 (Abcam Limited, UK), rabbit anti-Bax (Abcam), rabbit anti-cytochrome c (Abcam), rabbit anti-caspase-3 (Abcam), and rabbit anti-caspase-9 (Abcam). The samples were then incubated with second antibodies (anti-mouse HRP or anti-rabbit HRP antibody). After that the samples were incubated with Luminata™ Forte western HRP substrate (Millipore Sigma, Germany) for 5 min and the protein bands detected using a chemiluminescence scanner (C-DiGit; RICORbio Corporation, USA). The signal values ​​for each band were semi-quantitatively determined using Image Studio software (ver. 5.5.4, RICORbio).

### 2.11 Statistical analysis

All *in vitro* experiments (MTT viability assays, GC-MS volatile component quantification, western blotting, and Annexin V/PI, JC-1, and ROS assays) were performed using three independent biological replicates (n=3), except for GAPDH western blot quantification (n=5); each biological replicate represents an independently performed experiment conducted on a separate occasion. The *in vivo* study used 15 biologically independent mice per group (n=15). All data are expressed as mean ± standard deviation (SD) unless otherwise indicated.

For comparisons among three or more groups (MTT viability across essential oil or citral concentrations; GC-MS citral concentration and cell survival across proximal, middle, and distal well positions; western blot band intensity across control, 1.9 µg/mL, and 19.0 µg/mL citral treatment groups), one-way analysis of variance (ANOVA) followed by Bonferroni *post-hoc* multiple comparison testing was used, performed separately for each cell line and/or protein as applicable. For two-group comparisons of continuous variables (body weight, tumor area, and tumor weight between the M. salicifolia and control groups *in vivo*), one-way ANOVA (equivalent to an unpaired two-tailed t-test for two groups) with Bonferroni correction was used. For the comparison of tumor incidence (the proportion of tumor-bearing animals) between the M. salicifolia and control groups, Fisher’s exact test was used. Statistical significance was set at p<0.05. All statistical analyses were performed using GraphPad Prism version 9.4.1 (GraphPad Software, USA). Annexin V/PI, JC-1, and ROS assay results are presented as representative images from three independent experiments and were not subjected to formal statistical testing.

## 3 Results

### 3.1 Antitumor effect of *M. salicifolia* essential oil and citral in human breast cancer cells

To clarify the antitumor effects of the volatile components of *M. salicifolia* essential oil on murine breast cancer cell line 4T1 and human breast cancer cell line MCF-7, MTT assays were performed after 48 h of incubation (**Figure 1a**). Wells with actively proliferating cancer cells stained dark blue, whereas wells in which proliferation had ceased and cell death was induced turned yellow. In the undiluted *M. salicifolia* essential oil solution, cancer cells in all wells underwent cell death. This activity decreased in a distance-dependent manner as the *M. salicifolia* essential oil concentration decreased and almost disappeared at a 500-fold dilution (2.0 µg/mL) in both of 4T1 and MCF-7 cells (**Figure 1b**).

**Figure 2:**
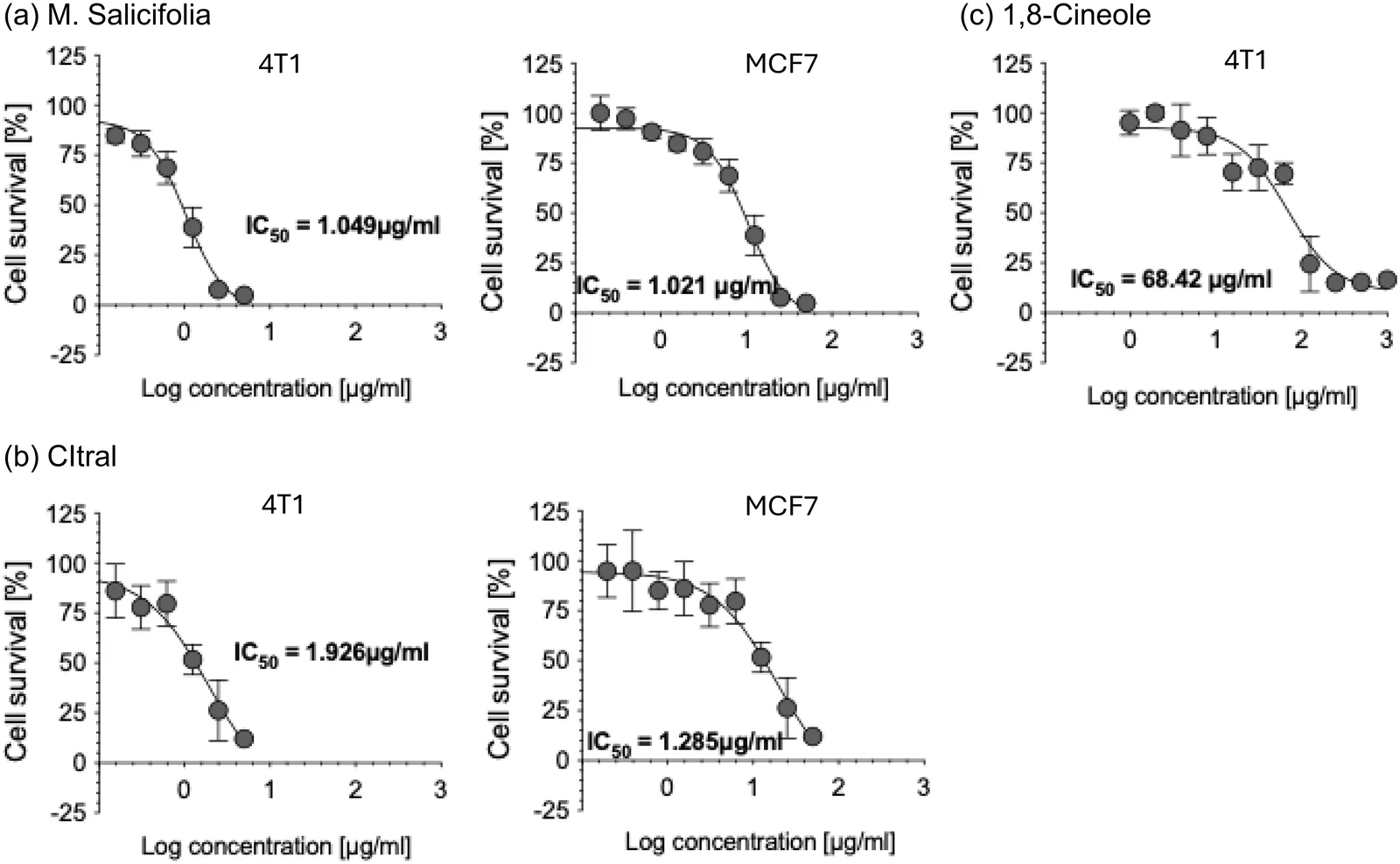
Tumor growth-inhibitory effect of diluted *Magnolia salicifolia* essential oil, citral and 1,8-Cineole. **(a)** Solutions of *M. salicifolia* essential oil diluted 200- (5 µg/mL) to 204,800-fold (0.0049 µg/mL) were directly treated4T1 and MCF-7 cells, and an MTT assay was performed after 48 h. Quantification of the tumor growth-inhibitory effect of *M. salicifolia* essential oil. The IC50 values ​​are expressed as the mean ± standard deviation (n=3, biological replicates). **(b)** Citral was diluted 5 µg/mL~0.0049 µg/mL were treated 4T1 and MCF-7 cells, and an MTT assay was performed (n=3, biological replicates). **(c)** 1,8-Cineole was diluted 5 µg/mL~0.0049 µg/mL were treated 4T1 cells and an MTT assay was performed (n=3, biological replicates).

To determine whether the antitumor activity of *M. salicifolia* essential oil extends to human breast cancer cells, we performed MTT assays using the human breast cancer cell line MCF-7. Both M. salicifolia essential oil and citral exhibited potent cytotoxic activity against MCF-7 cells, with IC50 values of 1.021 µg/mL and 1.285 µg/mL, respectively, comparable to those observed in 4T1 cells (IC50 = 1.05 µg/mL and 1.93 µg/mL, respectively) (**Figures 2a, b**, **Table 1**). These results demonstrate that the antitumor effects of *M. salicifolia* essential oil and citral are not restricted to murine breast cancer cells and are also observed in human breast cancer cells.

**Table 1:** IC50 values with 95% confidence intervals of *M.salicifolia*, citral, and 1,8-cineole.

Compound / cell line	IC50 (µg/mL)	95% CI (µg/mL)	R²	n
M. salicifolia essential oil / 4T1	1.05	0.729-1.511	0.984	3
M. salicifolia essential oil / MCF-7	1.021	0.863-1.179	0.984	3
Citral / 4T1	1.93	0.515-7.206	0.976	3
Citral / MCF-7	1.285	0.711-1.858	0.976	3
1,8-Cineole / 4T1	68.4	34.02-137.6	0.947	3

95% confidence intervals (CI) and goodness-of-fit (R²) for all IC50 values of M.salicifolia essential oil, citral and 1,8-cineole were presented, each based on n=3 independent biological replicates.

### 3.2 Antitumor effects of *M. salicifolia* essential oil *in vivo*

**Figure 3:**
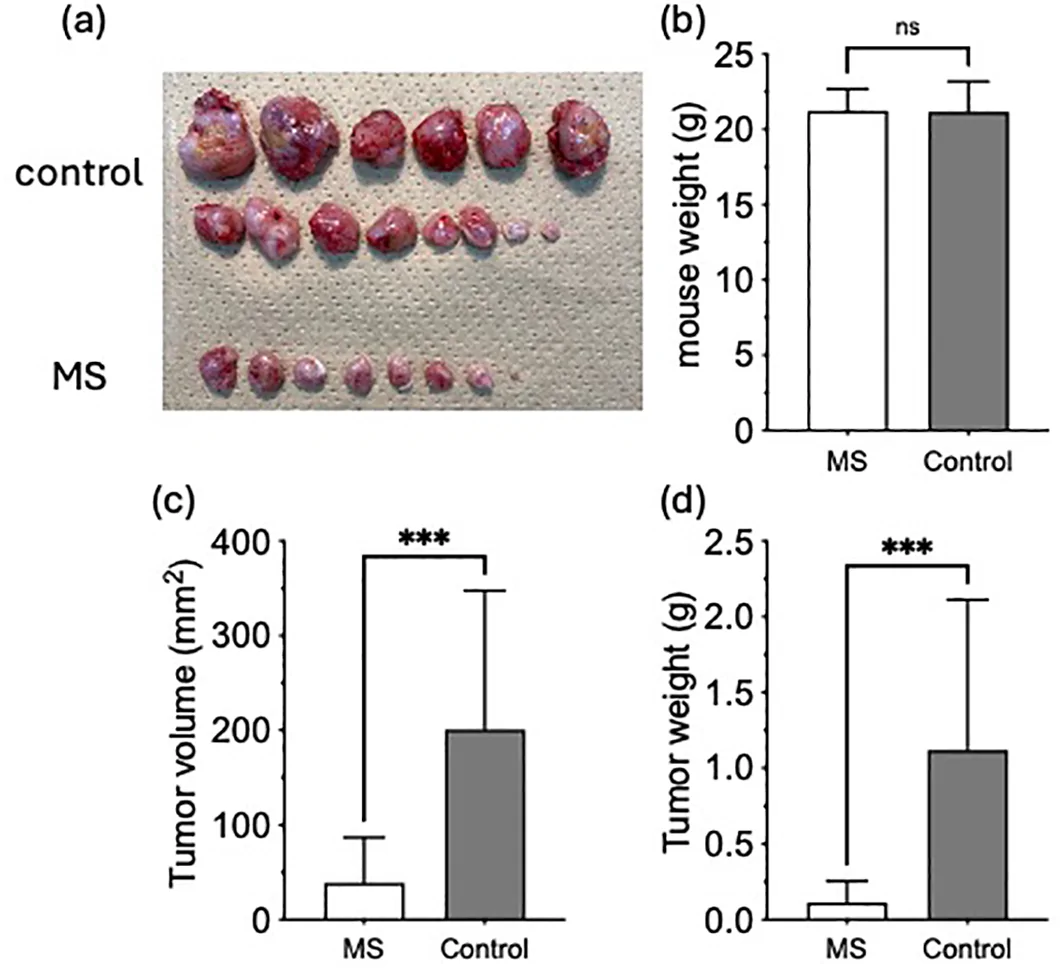
Antitumor effect of *Magnolia salicifolia* essential oil in a mouse model of metastatic breast cancer. **(a)** Murine 4T1 breast cancer cells were transplanted subcutaneously into 30 mice, of which 15 mice were exposed to the scent of *M. salicifolia* essential oil gas. Subcutaneous tumors were excised from the mice one month later, and the number of tumors was compared (control: control group, MS: *Magnolia salicifolia* group). Tumor incidence was compared between groups using Fisher’s exact test (p=0.0142). **(b)** The body weights of the mice were measured one month after tumor cell transplantation, and the average body weights (g) of the control and MS groups were compared. **(c)** Comparison of the surface areas (mm^2^) of the excised subcutaneous tumors. **(d)** Comparison of the weights (g) of the subcutaneous tumors. Body weight, tumor area, and tumor weight were compared between groups using one-way ANOVA with Bonferroni correction. ns: not significant, ***p<0.005; ns: no significant difference.

To investigate the effects of the volatile components of *M. salicifolia* essential oil *in vivo*, we conducted animal experiments using mouse models (**Figure 3**). The mice were housed in sealed containers for 8 h per day. After allowing *M. salicifolia* essential oil to vaporize naturally, the average citral concentration in the containers reached 4.31 µg/L. This confirmed that mice received a defined and reproducible airborne citral exposure dose; plasma and tissue citral concentrations were not measured in this study. In the control group, 14 of 15 mice (93%) had subcutaneous mammary cancer tumors, whereas in the group that inhaled the scent of *M. salicifolia* for 8 h per day, only 7 of 15 mice (47%) had subcutaneous tumors (**Figure 3a**). There were no changes in appetite or body weight in either group (**Figure 3b**). The *M. salicifolia* group (MS) showed a significant reduction in both the area and weight of the excised tumors compared with the control group (**Figures 3c, d**). Furthermore, no toxicity with body weight-loss was observed in mice following inhalation of the volatile components of *M. salicifolia* essential oil.

### 3.3 Chemical composition and role of citral for breast cancer cell

The constituent compounds of *M. salicifolia* essential oil were analyzed using GC-MS (**Table 2**). The results showed that it contained high concentrations of 1,8-cineole (18.50%), neral (10.66%), geranial (15.09%) and trans-anethole (9.99%). To compare the antitumor effects of citral (a *cis*- and *trans*-mixture) and 1,8-cineole, which are abundant in *M. salicifolia* essential oil, standards of these compounds were applied to 4T1 cells, and an MTT assay was performed after 48 h. The tumor growth-inhibitory effects of citral and 1,8-cineole were quantified, and their IC50 values ​​were determined. The IC_50_ value of citral was 1.93 µg/mL, while that of 1,8-cineole was 68.4 µg/mL, which was 35.5 times higher than that of citral (**Figure 2c**, **Table 2**). Furthermore, comparing the IC50 values ​​of each component multiplied by their concentrations in the essential oil, that of citral was 0.5, while that of 1,8-cineole was 12.7, indicating that 1,8-cineole was 25.5 times less potent than citral. These results show that citral exhibited cytotoxicity than 1,8-cineole, indicating that citral was the primary active compound in *M. salicifolia* essential oil.

**Table 2:** Component analysis of *Magnolia salicifolia* essential oil.

Area%	Name	R.Time
18.50	1,8-Cineole	19.471
15.09	Geranial	35.684
10.66	Neral	33.605
9.99	trans-Anethole	36.929
5.70	para-Cymene	18.896
5.26	Sabinene	15.642

The constituent elements of *M. salicifolia* essential oil were analyzed using gas chromatography. The components present in the essential oil at a concentration of 5% or more are shown (R.TIME: retention time).

### 3.4 Distance-dependent antitumor effects of the volatile components of *M. salicifolia* essential oil

**Figure 4:**
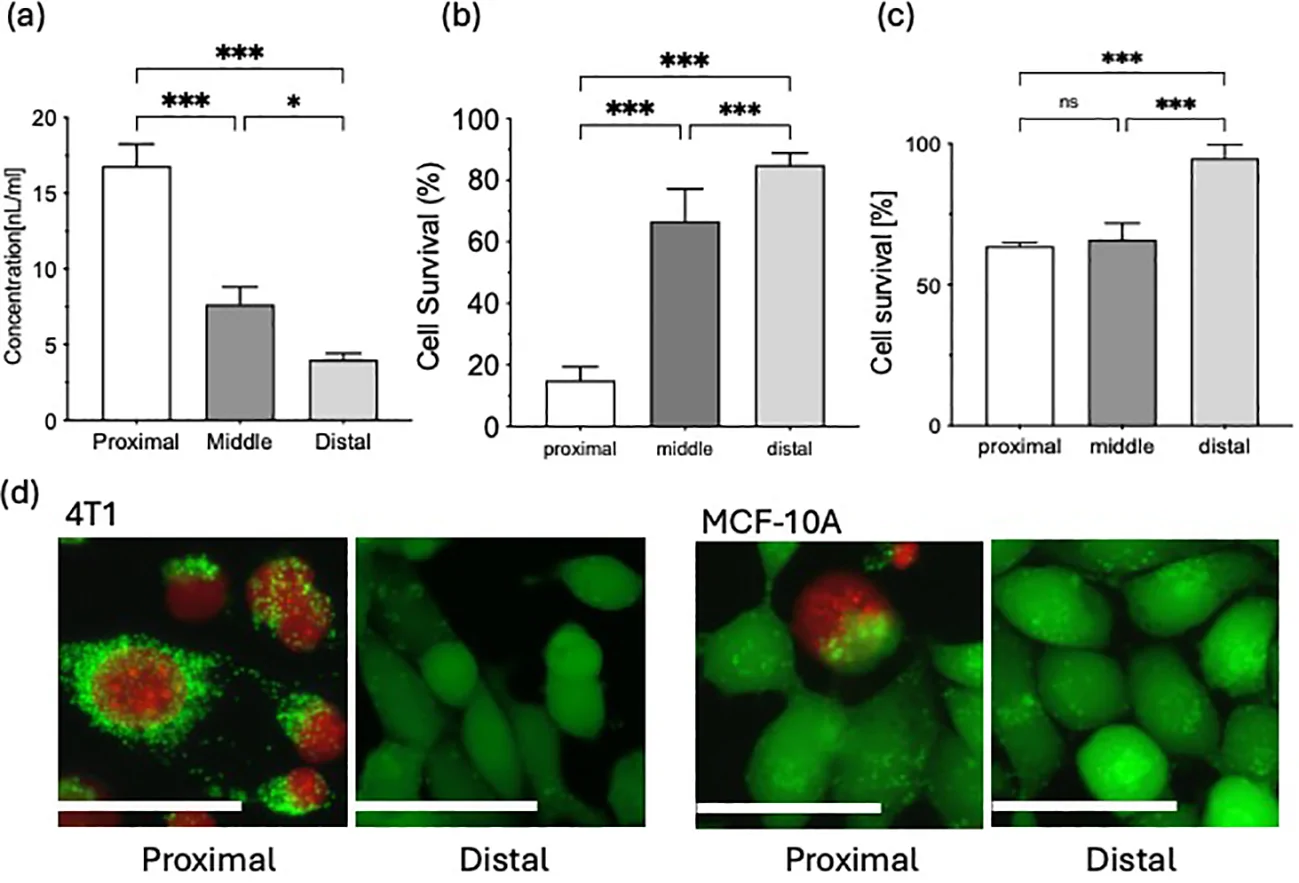
Relationship between distance from *Magnolia salicifolia* essential oil, concentration of vaporized citral, and antitumor effects. **(a)** Relationship between the distance between the essential oil and citral concentration. MonoTraps (n=3) were placed in each well (proximal: 0–0.8 cm, middle: 0.8–1.6 cm, distal: 1.6–2.4 cm) away from 50 µL of *M. salicifolia* essential oil (1000 µg/mL). After 48 h, the concentration of citral vapor absorbed by the cell monolayer in each well was measured via gas chromatography (n=3, biological replicates). **(b)** Effect of distance from the *M. salicifolia* essential oil and cancer cell survival rate. An MTT assay was performed using *M. salicifolia* essential oil diluted to 3.3 µg/ml to investigate the relationship between the distance from the essential oil to each well and cancer cell survival rate (n=3, biological replicates, *P < 0.05, ***P < 0.005). **(c)** Effect of distance from *M. salicifolia* essential oil to the MCF-10A normal breast cell survival. An MTT assay was performed using *M. salicifolia* essential oil diluted to 3.3 µg/ml and MCF-10A cell survival rate was shown (n=3, biological replicates, ns: P >0.05, ***P < 0.005). **(d)** Changes in 4T1 cancer cells and MCF-10A normal breast cells that was treated the volatile components of *M. salicifolia* essential oil was observed via fluorescence microscopy. Figures of each cells in proximal/distal wells were shown. Living cells were fluorescently stained green (calcein-AM) and the nuclei of dead cells were fluorescently stained red (PI). White line: 50 µm.

To determine whether the antitumor effect of *M. salicifolia* essential oil is selective for cancer cells, MTT assays were performed using *M. salicifolia* essential oil diluted to 3.3 µg/mL, and the survival rates of 4T1 breast cancer cells and MCF-10A non-tumorigenic breast epithelial cells were compared at each distance from the essential oil (**Figures 4c, d**). The concentration of citral decreased significantly (p<0.05) in the wells farther from the essential oil (**Figure 4a**), and the survival rate of 4T1 cancer cells in these wells increased (**Figure 4b**). In contrast, MCF-10A cells retained high viability (≥60% survival in the proximal wells), and no significant reduction in survival was observed in the proximal or middle wells (**Figure 4c**). Fluorescence microscopy confirmed that cells in the proximal wells exhibited cell shrinkage and nuclear aggregation characteristic of cell death in 4T1 cells, whereas no comparable changes were observed in MCF-10A cells (**Figure 4d**). These results indicate that the volatile components of *M. salicifolia* essential oil selectively induce cell death in cancer cells while largely sparing normal breast epithelial cells.

### 3.5 Mechanism of action of *M. salicifolia* essential oil

**Figure 5:**
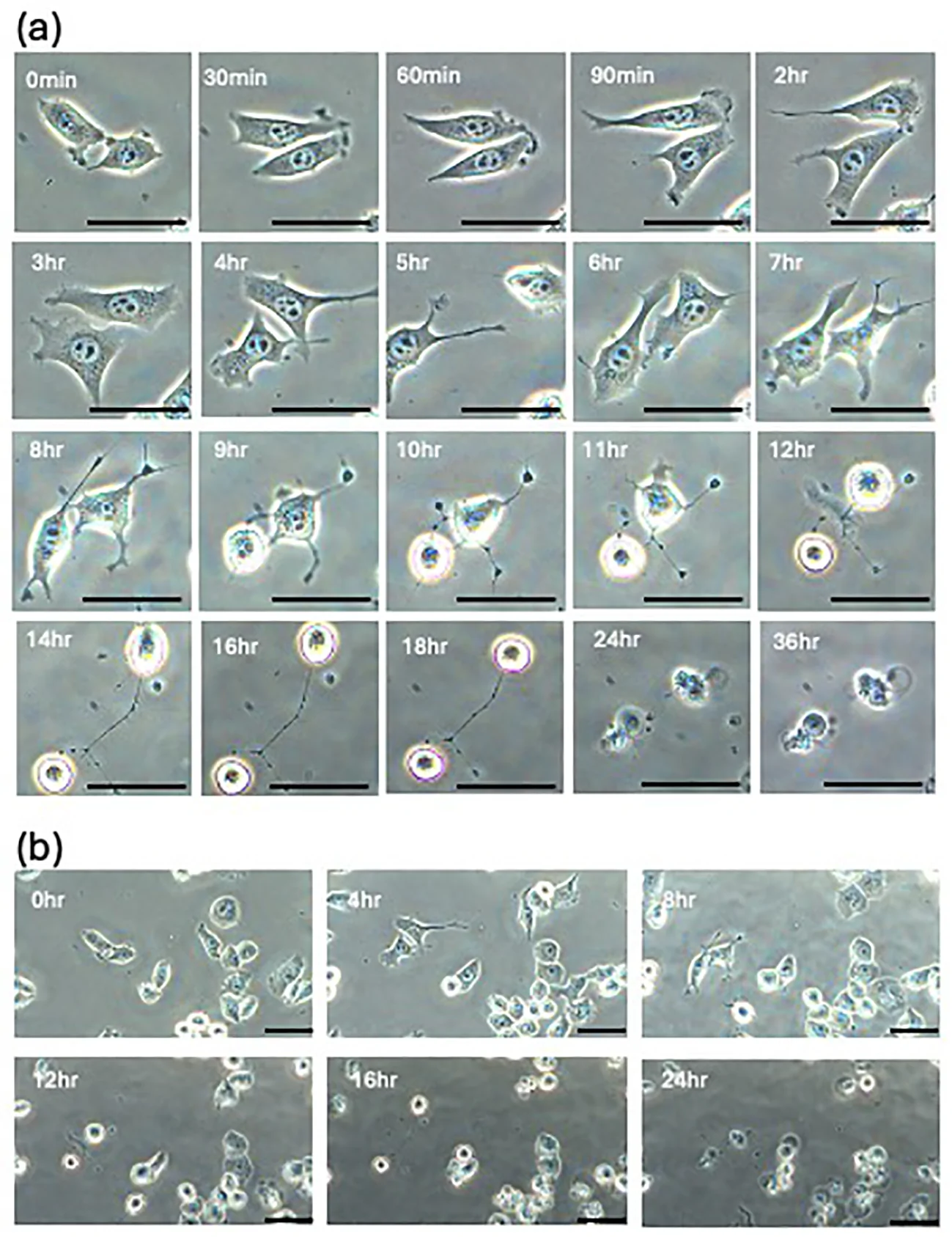
Time course changes in 4T1 breast cancer cells after citral administration. **(A)** Citral equivalent to the IC_50_ concentration was added to 4T1 cells and the time course of the morphological changes in the cells was observed using a light microscope (200× magnification). Black line: 50 µm. **(B)** Movement of the 4T1 cell population observed under a light microscope (100× magnification). Black line: 50 µm.

Previous results have shown that citral is the most abundant component in *M. salicifolia* essential oil and that it has potent antitumor effects. When 4T1 cells were treated with citral at the IC_50_, they initially underwent active morphological changes, migrated, and adhered to other cells (**Figure 5a**). However, after approximately 12 h, their movement slowed, cell adhesion decreased, and they became spherical. After 16 h, their movement almost completely ceased, and they lost their adhesion and began to float (**Figure 5b**).

**Figure 6:**
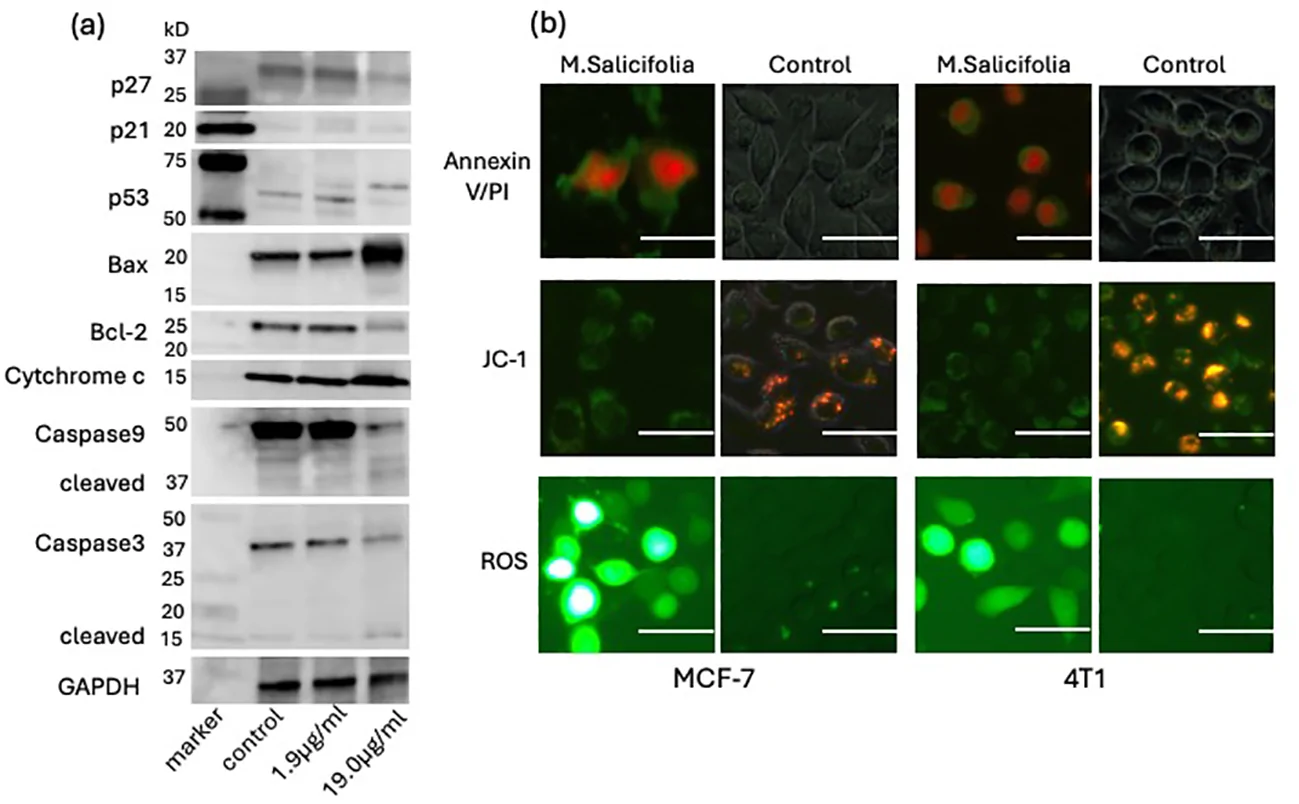
Citral-induced mitochondria-mediated apoptosis in breast cancer cells. **(a)** 4T1 cells were treated with citral at concentrations of 1.9 µg/mL and 19.0 µg/mL, corresponding to the IC50 values, for 14 h; untreated cells served as controls. Whole-cell protein lysates were analyzed by western blotting for p27, p21, p53, Bcl-2, Bax, cytochrome c, caspase-9 (full-length and cleaved), caspase-3 (full-length and cleaved), and GAPDH (loading control). Molecular weight markers (kDa; Precision Plus Protein Kaleidoscope Standards, Bio-Rad) are indicated alongside each blot. Uncropped, full-membrane images are provided in **Supplementary Figure 1**. Semi-quantitative densitometric values for each band are shown in **Table 2** (n=3, biological replicates). **(b)** 4T1 and MCF-7 cells were exposed to M. salicifolia essential oil (M. Salicifolia) or left untreated (Control) and stained with Annexin V-FITC/PI (apoptosis detection kit, Dojindo; green: Annexin V-FITC, red: PI), JC-1 (MitoMP Detection Kit, Dojindo; orange/red: J-aggregates in polarized mitochondria, green: monomers in depolarized mitochondria), or DCFH-DA (ROS Assay Kit–Highly Sensitive, Dojindo; green: intracellular ROS). Representative fluorescence images are shown for MCF-7 and 4T1. White scale bars: 50 µm.

Western blotting was then performed to analyze the changes in protein expression. Citral was added to 4T1 cells at concentrations of 1.9 µg/mL and 19.0 µg/mL, corresponding to the IC_50_ values of its components, and the cells were allowed to react for 14 h. After treatment, total protein was extracted, and western blotting was performed (**Figure 6a**, **Table 3**). The results showed that treatment with 19.0 µg/mL citral induced the cleavage of caspase-3 and capase-9, and the levels of these proteins decreased. Furthermore, Bax levels increased approximately 2.4-fold (798,000 ± 15,000 vs. 337,000 ± 96,000 in controls, p<0.001) and cytochrome c levels increased approximately 1.6-fold following treatment with 19.0 µg/mL citral. In contrast, Bcl-2, which is thought to have an inhibitory effect on cell death, decreased approximately 2.3-fold (34,450 ± 3,850 vs. 78,700 ± 1,700 in controls, p<0.001). Expression of p53, p21, and p27, which are involved in cell cycle regulation, slightly decreased. Therefore, citral may act by increasing Bax and decreasing Bcl-2 at the mitochondrial membrane of cancer cells, thereby lowering the mitochondrial membrane potential and increasing cytochrome c levels, consistent with activation of the mitochondrial apoptotic pathway, and activating caspase-3 and caspase-9.

**Table 3:** Signal intensity values of each western blot band (semi-quantitative).

Protein	n	Mean ± SD, p-factor		
		Control	1.9µg/ml	19.0µg/ml
P27	3	22400±6100	17150±3050	1817±1423**
P21	3	3485±1125	3890±390	1058±62**
P53	3	4775±725	5025±1915	357±126**
Bax	3	337000 ± 96000	329000 ± 13000	798000 ± 15000***
Bcl2	3	78700 ± 1700	68700±2900*	34450 ± 3850**
Cytchrome C	3	235500±5500	235000±7000	383000±1000***
Caspase 9	3	279500±39500	270000±27000	185950±85050
Cleaved Cas9	3	2820±1420	3065±1205	3870±360
Caspae 3	3	64150±25950	36000±15700	17100±8800*
Cleaved Cas3	3	5783±5317	4136±3534	9682±8818
GAPDH	5	6974±2434	6850±2626	5202±2516

The signal intensity values of each band in the western blot were quantified using Image Studio software. The data for each band is shown as the signal value ± standard deviation. n: number of experiments performed.

### 3.6 Additional evidence confirming mitochondria-mediated apoptosis

To further substantiate the apoptotic mechanism suggested by western blot analysis, we performed Annexin V/PI staining, JC-1 mitochondrial membrane potential assay, and intracellular ROS measurement in 4T1 and MCF-7 cells exposed to *M. salicifolia* essential oil (**Figure 6b**). The proportion of Annexin V/PI-positive cells increased in both 4T1 and MCF-7 cells following exposure to *M. salicifolia* essential oil compared with untreated controls, confirming induction of apoptosis. JC-1 staining showed a shift from red to green fluorescence in treated cells, indicating a decrease in mitochondrial membrane potential consistent with mitochondrial depolarization. In addition, intracellular ROS levels were increased in treated cells relative to controls. Together, these findings corroborate the western blot data and support a mitochondria-mediated, ROS-associated apoptotic mechanism underlying the antitumor activity of M. salicifolia essential oil.

## 4 Discussion

Terpenoid phytochemicals have attracted considerable attention as naturally occurring anticancer agents because many members of this chemical class suppress malignant cell proliferation through common mechanisms, including induction of oxidative stress, mitochondrial dysfunction, and activation of the intrinsic apoptotic pathway. Recent comprehensive reviews have highlighted that these biological effects are shared across a wide variety of mono-, sesqui-, and diterpenoids, supporting the concept that terpenoids represent an important source of anticancer lead compounds (23). In agreement with this concept, our findings demonstrate that citral, a major monoterpenoid constituent of *Magnolia salicifolia* essential oil, induces mitochondria-mediated apoptosis characterized by mitochondrial membrane depolarization, increased cytochrome *c* levels, and activation of caspase-3 and caspase-9. The present study addressed the limited understanding of the antitumor activity of *M. salicifolia* essential oil and its volatile constituents. To fill this knowledge gap, we investigated its effects on breast cancer cells and explored the molecular mechanisms underlying its anticancer activity. Unlike previous studies that primarily investigated purified terpenoids by direct application to cultured cells, the present study further demonstrates that volatile terpenoids retain their biological activity under distance-dependent exposure conditions and exert antitumor effects *in vivo* following inhalation. These findings extend the current understanding of terpenoid pharmacology by highlighting the therapeutic potential of volatile phytochemicals administered through inhalation.

We previously reported that the volatile components of lemongrass, lemon myrtle, litsea, and melissa exhibit strong antitumor effects against breast cancer cells (12). Among these, lemon myrtle, which had the highest citral content, showed the most potent antitumor effect among the essential oils investigated. The citral content of the *M. salicifolia* essential oil reported here was 25.75%, which is approximately 30% of that in lemon myrtle, which contains 83.77% citral. However, the volatile components of *M. salicifolia* essential oil (IC_50_ = 1.05 µg/mL) showed a potent antitumor effect similar to that of lemon myrtle (IC_50_ = 1.26 µg/mL). Carvacrol, another component of *M. salicifolia* essential oil, has been reported to synergistically induce tumor cell death in acute myeloid leukemia cell lines (KG1, HL60, and K562) by activating apoptosis, oxidation, and reticular stress pathways in combination with thymol (24). *M. salicifolia* essential oil contains more than 50 components in addition to carvacrol (25), and these interactions reportedly exert a potent antitumor effect (26); therefore, further research is required to elucidate the antitumor mechanism of *M. salicifolia* essential oil.

The aromatic components of essential oils bind to olfactory cilia in the nasal cavity and stimulate olfactory cells, transmitting signals to the cerebrum via the olfactory nerve and exerting various effects via the sympathetic and parasympathetic nervous systems (27–29). In addition to olfactory stimulation, volatile aromatic compounds inhaled into the respiratory tract may be absorbed across the alveolar epithelium, potentially allowing them to reach target tissues and exert biological effects (30, 31). We hypothesize that the antitumor effect of M. salicifolia essential oil observed *in vivo* may be mediated, at least in part, by systemic absorption of citral from the lungs into the bloodstream and its subsequent delivery to tumor tissue. However, this study did not include pharmacokinetic or biodistribution analyses, and this proposed route therefore remains a hypothesis supported by indirect (exposure-dose and pharmacodynamic) evidence rather than a directly demonstrated mechanism (see Study Limitations). If confirmed, systemic circulation of volatile compounds after inhalation could allow them to modulate not only tumor cells directly but also components of the tumor microenvironment (32–34). This raises the possibility that inhalable phytochemicals can modulate immune responses and contribute to anticancer effects *in vivo*.

Mechanistically, our findings are consistent with those of previous reports showing that citral induces mitochondrial apoptosis (35, 36). Our measured citral IC50 values (1.93 µg/mL in 4T1; 1.285 µg/mL in MCF-7) are notably lower (i.e., citral was more potent) than several previously reported values: Chaouki et al. reported an IC50 of 180 µM (≈27.4 µg/mL) for citral in MCF-7 cells (36); Nigjeh et al. reported an *in vitro* IC50 of 9.0 µg/mL for citral in 4T1 cells; and a citral-loaded nanostructured lipid carrier study reported a free-citral IC50 of 21.4 ± 2.82 µg/mL in 4T1 cells (9). We discuss possible reasons for this difference in potency, including differences in the cis/trans citral isomer ratio and purity of the citral preparation, cell passage number, treatment duration, and MTT assay protocol details, and note that our findings are directionally consistent with the broader literature demonstrating citral’s antiproliferative/pro-apoptotic activity in breast cancer cells. In this report, the minimal change observed in the expression of cell cycle regulators suggests that apoptosis induction was the dominant cell death mechanism in this study. Importantly, these findings can be interpreted within the broader framework of cancer biology. Mitochondria-mediated apoptosis is a central mechanism of cancer cell death, and mitochondrial dysfunction has been recognized as a key therapeutic target in oncology (37). The activation of the Bax-cytochrome c-caspase axis observed in this study was consistent with this canonical pathway, further supporting the biological relevance of citral-induced apoptosis. It should be noted that cytochrome c was quantified in whole-cell lysates in this study; direct confirmation of cytochrome c translocation from the mitochondria to the cytosol via subcellular fractionation was not performed and is identified as a priority for future work (see Study Limitations). These conclusions were further corroborated by Annexin V/PI, JC-1, and intracellular ROS assays, which independently confirmed increased apoptosis, mitochondrial membrane depolarization, and oxidative stress in M. salicifolia essential oil-treated cells, providing converging, multi-assay support for a mitochondria-mediated apoptotic mechanism (**Figures 6a, b**). Furthermore, the comparable cytotoxic activity of *M. salicifolia* essential oil and citral observed in the human breast cancer cell line MCF-7 supports the relevance of these findings beyond a single murine model and strengthens the translational potential of this study. Although this study was conducted primarily *in vitro*, volatile compounds such as those derived from *M. salicifolia*, may hypothetically influence not only tumor cells but also the tumor microenvironment through systemic absorption; direct evidence for this route of action is discussed in the Study Limitations. Moreover, the preferential cytotoxicity of *M. salicifolia* essential oil toward 4T1 cancer cells relative to the non-tumorigenic MCF-10A mammary epithelial cell line suggests a favorable therapeutic window and supports its potential safety as an inhalable anticancer agent, although a formal, dose-response-based selectivity index has not yet been established.

## 5 Study limitations and translational potential

Several limitations of this study should be acknowledged. First, the *M. salicifolia* essential oil used here was obtained from a single commercial harvesting stage and processing batch; because the relative proportions of citral, 1,8-cineole, and trans-anethole in *Magnolia* essential oils are known to vary with harvest season, plant maturity, and geographic origin, the antitumor potency and chemical fingerprint reported here may not be fully representative of the species across harvests, and future studies should compare essential oils obtained from multiple harvesting stages and production batches. Second, our mechanistic (apoptosis pathway) and *in vivo* antitumor data were generated using a single murine mammary carcinoma cell line, 4T1. Although 4T1 is a well-established, immunocompetent-compatible model of triple-negative breast cancer, in response to reviewer comments we confirmed that *M. salicifolia* essential oil and citral also exert comparable cytotoxic effects in the human breast cancer cell line MCF-7 (IC50 = 1.021 and 1.285 µg/mL, respectively), supporting the translational relevance of our findings. However, the detailed apoptotic mechanism (mitochondrial membrane potential, cytochrome c release, and caspase-3/9 activation) was characterized only in 4T1 cells, and validation of this mechanism in human cell lines, extension to additional human breast cancer cell lines representing diverse molecular subtypes (e.g., triple-negative MDA-MB-231, hormone receptor-positive T47D, and HER2-positive lines), and confirmation of *in vivo* efficacy in human xenograft models will be required before the generalizability of these findings can be established. Third, the *in vivo* inhalation protocol employed here was limited to 8 h of exposure; the long-term efficacy, optimal exposure duration, and chronic safety of essential oil inhalation, including any cumulative pulmonary or hepatic effects, remain to be determined in extended dosing studies. Fourth, although *M. salicifolia* essential oil showed preferential cytotoxicity toward 4T1 cancer cells over the non-tumorigenic MCF-10A mammary epithelial cell line in a distance-dependent volatile exposure model, these data were not derived from a direct concentration-response assay, precluding calculation of a conventional IC50-based selectivity index. Direct dose-response comparison of *M. salicifolia* essential oil and citral in MCF-10A and additional non-tumorigenic cell lines will be required to formally quantify cancer selectivity and further support the therapeutic safety of this approach. Fifth, cytochrome c was assessed by western blotting of whole-cell lysates in this study. Although the observed increase in cytochrome c levels is consistent with activation of the mitochondrial apoptotic pathway and aligns with the concurrent changes in Bax, Bcl-2, and caspase-3/9, definitive demonstration of cytochrome c release requires separate analysis of mitochondrial and cytosolic protein fractions, which was not performed here. Subcellular fractionation to directly confirm cytochrome c translocation from the mitochondria to the cytosol is planned for future work. Sixth, although the *in vivo* inhalation experiments demonstrated a reproducible airborne citral exposure dose (mean container concentration of 4.31 µg/L) and a corresponding reduction in tumor incidence and burden, no pharmacokinetic or biodistribution studies (e.g., measurement of citral concentrations in plasma, lung, or tumor tissue) were performed to directly demonstrate systemic absorption or tumor accumulation of citral following inhalation. The proposed mechanism of systemic delivery therefore remains a plausible hypothesis supported by indirect exposure-dose and pharmacodynamic evidence rather than a directly confirmed pharmacokinetic finding. Future studies incorporating plasma and tissue citral quantification (e.g., by LC-MS/MS) following inhalation exposure will be required to formally establish systemic absorption and tumor biodistribution. Seventh, the volatile exposure model used in this study relied on a standard, unmodified cell culture incubator (37 °C, 5% CO2) without independent control or measurement of air exchange rate or humidity specific to the sealed exposure system, and citral concentration was quantified only as a single endpoint measurement after 48 h rather than as a time-resolved profile of evaporation kinetics. Although GC-MS quantification (n=3) and MTT viability assays (n=3) demonstrated a consistent and reproducible distance-dependent exposure-response relationship, future studies incorporating controlled air exchange and humidity and continuous or time-course headspace GC-MS sampling will be needed to fully characterize the physical parameters governing this exposure model and to more precisely estimate the time-integrated exposure dose. Eighth, several aspects of the *in vivo* study design should be noted to aid interpretation of its rigor and reproducibility. Although mice were randomly allocated to groups, outcome assessment was not performed in a blinded manner, which is a potential source of observer bias. The sample size (n=15 per group) was not derived from a formal *a priori* power calculation. Tumor area and weight were measured only once, at the study endpoint, rather than longitudinally over the course of the experiment. Predefined humane endpoints, in accordance with our institutionally approved protocol (Dosho 23-552), were applied throughout the study. Future *in vivo* studies will incorporate blinded outcome assessment, formal sample size calculations, and serial in-life tumor measurements to further strengthen experimental rigor.

Despite these limitations, the present findings support further exploration of inhalable, volatile essential oil constituents as a novel class of adjuvant anticancer aromatherapy agents. Because inhalation delivery bypasses first-pass hepatic metabolism and avoids the injection-site and gastrointestinal morbidity associated with conventional chemotherapy, it may be particularly well suited as a low-burden complementary intervention for elderly or frail patients, or as a maintenance strategy between cytotoxic treatment cycles. Realizing this translational potential will require several parallel lines of investigation: pharmaceutical strategies, such as nanostructured lipid carriers or other co-assembly and encapsulation technologies, to stabilize volatile terpenoids and sustain therapeutic-range systemic exposure and bioavailability (38); standardization of essential oil composition and dosing, given the chemotype variability noted above; combination studies evaluating whether inhaled citral-rich essential oils can be safely co-administered with standard endocrine, cytotoxic, or immune checkpoint-based therapies without adverse pharmacokinetic interaction; and, ultimately, early-phase clinical studies incorporating both objective tumor response measures and patient-reported outcomes such as symptom burden and quality of life, which represent a central rationale for pursuing this therapeutic strategy. Addressing these questions will help clarify whether volatile phytochemical inhalation can move from a promising preclinical concept toward a clinically viable adjunct in breast cancer care.

In addition, although Annexin V/PI, JC-1, and intracellular ROS assays provided converging support for the proposed mitochondria-mediated apoptotic mechanism, caspase inhibition (rescue) experiments and TUNEL staining were not performed in the present study. Caspase inhibitor studies would help establish a causal requirement for caspase activity in M. salicifolia essential oil-induced cell death, and TUNEL staining would provide additional single-cell confirmation of apoptotic DNA fragmentation; both are identified as priorities for future work.

## 6 Conclusion

**Figure 7:**
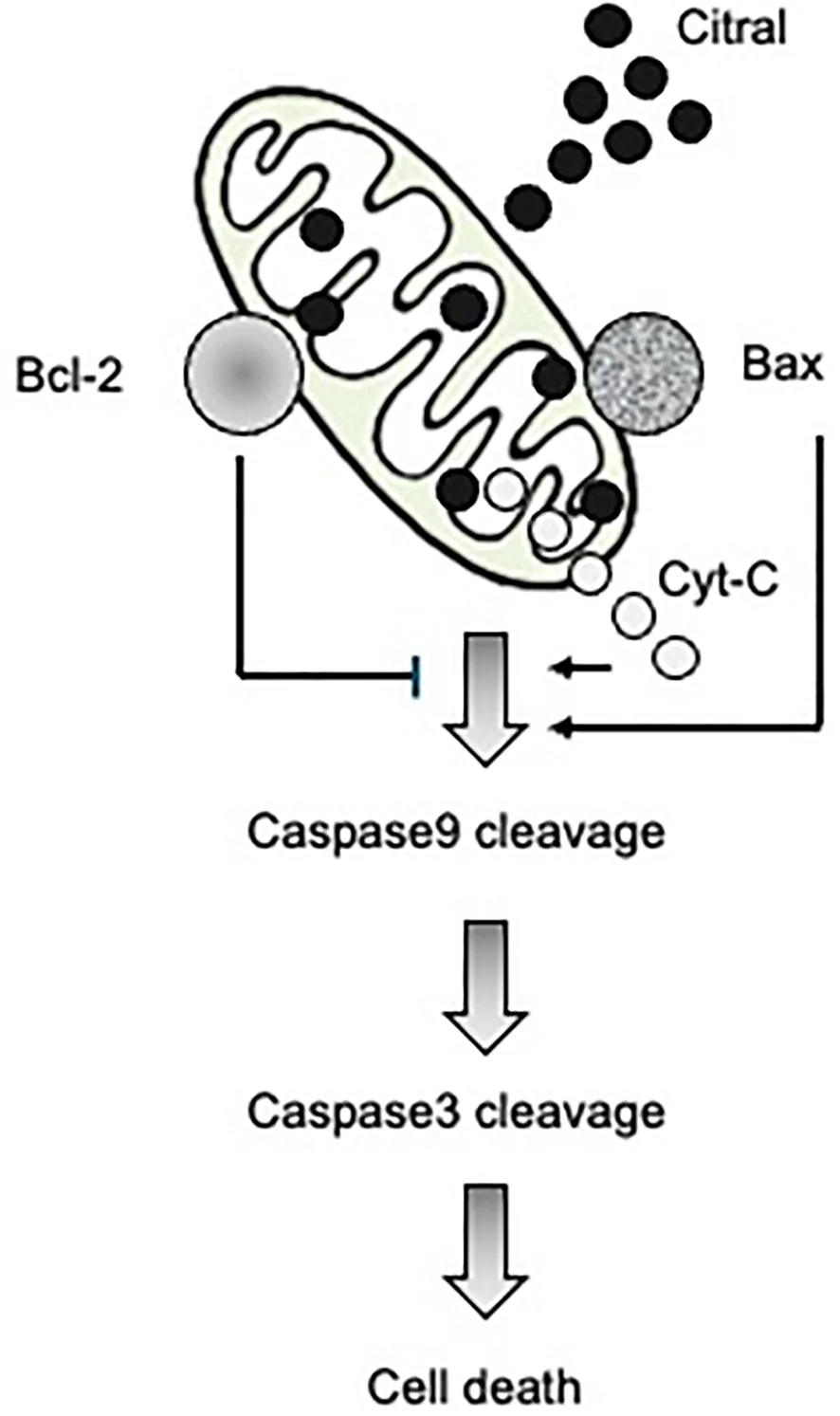
Proposed mechanism of citral-induced mitochondria-mediated apoptosis. Hypothesis of the antitumor mechanism of citral. Cyt-C, cytochrome c.

In conclusion, our results showed that *M. salicifolia* essential oil exhibited antitumor effects against 4T1 mouse mammary cancer cells, with an IC_50_ of 1.05 μg/mL. The active antitumor compounds of *M. salicifolia* essential oil were found in its volatile components, whose effects decreased in a distance-dependent manner *in vitro*. Our analyses also revealed that *M. salicifolia* essential oil contains many low-molecular-weight compounds, such as 1,8-cineole, neral, geranial, and transanethole. Finally, the mechanism by which *M. salicifolia* involves increased cytochrome c levels and activation of caspase-3 and caspase-9, following a Bax/Bcl-2-mediated decrease in mitochondrial membrane potential, consistent with mitochondria-mediated apoptosis (**Figure 7**).

## Data Availability

The datasets presented in this study can be found in online repositories. The names of the repository/repositories and accession number(s) can be found in the article/**Supplementary Material**.
